# Effects of patulin on quail muscle cells and the potential for microbiome-mediated recovery

**DOI:** 10.1016/j.psj.2025.105872

**Published:** 2025-09-19

**Authors:** Jeong woong Park, Hana Kim, Seon-Ae Choi, Hak Kyo Lee, Dong Hyun Shin

**Affiliations:** a Department of Animal Biotechnology, Jeonbuk National University, Jeonju 54896, South Korea; b Center for Industrialization of Agricultural and Livestock Microorganisms (CIALM); c Department of Agricultural Convergence Technology, Jeonbuk National University, Jeonju 54896, South Korea; d The Korean Society of Animal Big Data Research, Korean Society of Animal Science and Technology, Seoul 06367, South Korea

**Keywords:** Mycotoxin, Quail muscle cells, injury recovery, *Bacillus subtilis*, *Bacillus velezensis*

## Abstract

Mycotoxins pose a significant threat to livestock health and productivity by compromising immunity and inducing various toxicities. This study investigated the potential of specific *Bacillus* strains to mitigate mycotoxin-induced muscle damage in poultry cells. We treated quail muscle clone 7 (QM7**)** muscle cells with patulin, a common mycotoxin, to induce cellular injury. Subsequently, the damaged QM7 cells were treated with the candidate microbial strains, *B. subtilis* and *B. velezensis*. Our findings revealed that patulin treatment elevated stress-inducible gene expression and apoptosis markers, concurrently disrupting normal myoblast differentiation, as evidenced by altered expression patterns of Paired Box 7 (*PAX7*) and Myogenic Differentiation 1 (*MyoD*) and impaired myotube formation. Notably, treatment with the *Bacillus* strains significantly reduced these negative effects, reducing stress and apoptosis indicators while promoting a different pattern of myotube development. Although the exact mechanism of muscle recovery warrants further functional assessment, our results highlight the potential of *B. subtilis* and *B. velezensis* as agents for mitigating mycotoxin-induced damage in poultry, and offer novel strategies for enhancing animal health and agricultural sustainability.

## Introduction

Poultry is indispensable to global food security as a cost-effective source of protein. In particular, in developing countries, poultry serves as a primary source of animal protein (Bagust, 2013). The poultry industry, which encompasses the breeding and production of various poultry species such as chickens, ducks, and geese for human consumption, is a global industry. Consequently, the poultry industry has consistently expanded, reaching a global market value of $385 billion. With the steady growth of the poultry industry, research and development have been actively conducted in various fields of poultry science. Genetic studies are being conducted for improving disease resistance (Idoko-Akoh et al., 2023), growth rate, and feed efficiency (Jia et al., 2018). Furthermore, nutritional research focused on developing feed additives optimized for poultry growth and health (Pirgozliev et al., 2019) and studies on automation systems designed to enhance breeding environments (Neethirajan, 2022) are being conducted. Chicken meat production and skeletal muscle development are closely associated. Therefore, a comprehensive understanding of muscle proliferation, differentiation, and regeneration is critical to improve the yield and quality of chicken meat.

Muscle differentiation is defined as the process by which myoblast cells differentiate and subsequently fuse to form myotubes, and plays a crucial role in improving poultry breeding and productivity. Muscle differentiation is mediated by the regulation of various genes (Molkentin and Olson, 1996) and complex cell signaling pathways (Bakkar and Guttridge, 2010; Keren et al., 2006; von Maltzahn et al., 2012). Myoblasts are regulated by various factors including Myogenic Differentiation 1 (***MyoD***), Myogenic Factor 5 (***Myf5***), and myogenin (***MyoG***), which are key regulators collectively known as Myogenic Regulatory Factors (**MRFs**) (Molkentin and Olson, 1996). Moreover, the proliferation and differentiation inherent in muscle differentiation are regulated by diverse signaling pathways, including Wnt, Notch, Hedgehog, and transforming growth factor-beta (Bakkar and Guttridge, 2010; Keren et al., 2006; Molkentin and Olson, 1996; von Maltzahn et al., 2012).

Mycotoxins are secondary metabolites produced by filamentous fungi and are naturally occurring contaminants that can pollute various food crops (Bennett, 1987; Tanaka et al., 1988). Livestock, including poultry, can be exposed to mycotoxins via contaminated aged feed. This can lead to a reduction in animal immunity, an increased risk of disease, and decreased productivity (Bergsjø et al., 1993; Kipper et al., 2019). In severe cases, mycotoxins can induce various toxic effects, such as hepatotoxicity, teratogenicity, and mutagenicity (Alshannaq and Yu, 2017), and can cause diseases such as hepatitis, hemorrhage, edema, and cancer (Befa; Chhonker et al., 2018). The International Agency for Research on Cancer has classified mycotoxins as human carcinogens. These mycotoxins can accumulate in animal bodies and can be detected as residues in livestock products, such as meat, milk, and eggs (Richard, 2007). Management strategies for mycotoxins are used to establish proper feed storage and management methods to inhibit fungal growth, minimize mycotoxin production, safely dispose of feed contaminated with mycotoxins instead of feeding it to animals, and utilize additives that are effective in detoxifying mycotoxins (Atanda et al., 2011).

This study aimed to investigate the effects of mycotoxins on poultry muscle cell differentiation and reduce mycotoxin-induced damage using various natural products and microbiomes. We cultured poultry muscle cells and induced muscle cell damage by treating them with the mycotoxin patulin. Subsequently, we attempted to repair the mycotoxin-induced muscle damage by treating cells with various natural products and candidate microbiomes. This study suggests potential solutions for reducing livestock production losses caused by mycotoxin contamination of poultry and other animal feed.

## Materials and methods

### Cell culture and patulin treatment

Quail muscle clone 7 (**QM7**) cells were maintained and sub-passaged in Dulbecco’s Modified Eagle’s Medium (Gibco, Grand Island, NY, USA) supplemented with 10 % fetal bovine serum (**FBS**, Gibco, Grand Island, NY, USA) and 1 % antibiotic-antimycotic (penicillin-streptomycin [10,000 U/mL], Gibco, USA). The cells were cultured at 37°C in a humidified atmosphere containing 5 % CO_2_. The medium was changed thrice a week. Cells at 70–80 % confluency were gently washed twice with PBS and harvested using 0.25 % trypsin-EDTA (Gibco, Canada) for expansion. To induce myotube differentiation at 90 % confluency, the differentiation medium containing 0.5 % FBS and 1× antibiotic-antimycotic was changed, and half of the medium was replaced daily with fresh differentiation medium. Patulin was purchased from Sigma-Aldrich (St. Louis, MO, USA) and used to induce muscle damage. To induce patulin stimulus, QM7 cells were incubated at 80 % confluency and treated with 2.5 µg/mL of patulin for 3 days.

### RNA extraction and complementary DNA synthesis

QM7 cells from the culture were plated in a six-well plate and incubated for 24 hours. The cells were then treated with patulin (2.5 µg/mL) and incubated for 3 days and harvested. RNA was isolated using the RNeasy Plus Mini Kit (Qiagen, Hilden, Germany). Subsequently, 600 µL of RLT plus buffer was added to the harvested cell pellets and the mixture was vortexed for 30 sec thoroughly to ensure complete cell lysis. The mixture was then transferred to a spin cartridge with a collection tube and centrifuged at 12,000 × g for 30 s at 4℃. After centrifugation, the flow-through was saved, and 600 µL of 70 % ethanol was added to it. The mixture was then gently pipetted and transferred to a new spin cartridge. The mixed samples were then centrifuged at 12,000 × g for 15 sec and the flow-through was discarded. Subsequently, 700 µL of RW1 buffer was added to the spin cartridge and centrifuged at 12,000 × g for 15 s. The flow-through was discarded, and 500 µL of RPE wash buffer was added to the spin column. The sample was centrifuged at 12,000 × g for 15 sec and the flow-through was discarded and the spin cartridge was reinserted into the same collection tube. This process was repeated once and centrifuged at 12,000 × g for 2 mn to dry the membrane with the bound RNA. Subsequently, the flow-through was discarded, and the spin cartridge was inserted into a 1.5 mL recovery tube. Next, 30 µL of RNase free water was added to the center of the spin cartridge and centrifuged at 12,000 × g for 1 mn to elute the RNA from the membrane into the recovery tube. RNA quantity was determined using a spectrophotometer. RNA measurements were used to calculate the volume of RNA, H_2_O, and 5X PrimeScript RT Master Mix for cDNA synthesis. cDNA synthesis was conducted using the PrimeScript RT Master Mix (RR036A, Takara, Japan).

### Quantitative reverse transcription PCR (qPCR)

To quantify the gene expression levels of inflammatory marker genes and signaling cascade-located genes under patulin and patulin-microbiome co-culture stimuli, a qPCR was conducted using the BioRad CFX-96 apparatus (BioRad, Hercules, CA, USA). Sequence-specific primers (Table 1) were designed using the Primer-BLAST PRIMER3 software (http://bioinfo.ut.ee/primer3-0.4.0/). Each reaction was performed in a 20 µL mixture containing 10 µL of TB green Premix Ex Taq Ⅱ, 1 µL of forward primer (10 pmol), 1 µL of reverse primer (10 pmol), 0.4 µL of ROX reference Dye, 6.6 µL of distilled water, and 1 µL (200 ng/μL) of cDNA. PCR conditions were as follows: a pre-denaturation step at 94°C for 5 min; 39 cycles of 94°C for 30 s, 59°C for 30 s, and 72°C for 30 s; and a final step of 72°C for 10 min. All measurements were performed in triplicate for all specimens, and the 2 ^-ΔΔCT^ method was used to compare the data. The relative expression of each target gene was calculated by normalizing its expression to that of glyceraldehyde-3-phosphate dehydrogenase.

**Table 1:** Primers used in this study for qPCR analysis.

Primer name	Primer sequence (5′ to 3′)	Tm (°C)	Product size (bp)
ATF3 –F	AAGTGGTTCGGGAGTGATGT	**59**	148
ATF3 –R	TTCCTTCTCCTCTGGTGCTG		
PAX7 –F	GATCGTGGAAATGGCTCACC	**59**	106
PAX7 –R	CGTCTCTTGGTACCTGCAGA		
BCL2 –F	ATGACCGAGTACCTGAACCG	**59**	169
BCL2 –R	CAAGAGTGATGCAAGCTCCC		
FOXO1 –F	AAGCCCCAGCTCTCACAGTA	**60**	203
FOXO1 –R	TCTCTGAAAGGCTGGGAAGA		
MAFbx –F	GGCTGCTGTGGAAGAAACTC	**58**	188
MAFbx –R	CCAAGAGAGGATGTGGCAAT		
MyoD –F	CTGTTGTTTCCAGCCTCGAC	**59**	157
MyoD –R	ATCTGGGCTCCACTGTCATT		
Desmin –F	TATCGCCACCAGATCCAGTC	**59**	165
Desmin –R	TTTCAAGTGCCGGATCTCCT		
CASP3 –F	TGGTACAGATGGCCCTCTTG	**59**	158
CASP3 –R	TTTCATCTGGTCCGCTGTCT		
GAPDH –F	CTGGGAAGTTGTGGAGGGAT	**59**	167
GAPDH –R	GCAGGTCAGGTCAACAACAG		

### Statistical analysis

Both t-tests and ANOVA were conducted to determine the significance. Data are shown as the mean ± standard deviation. Duncan’s multiple range test followed by one-way ANOVA were used for comparisons among different incubation times in each group.

## Results

### Patulin-induced muscle damage in QM7 cells

**Fig. 1:**
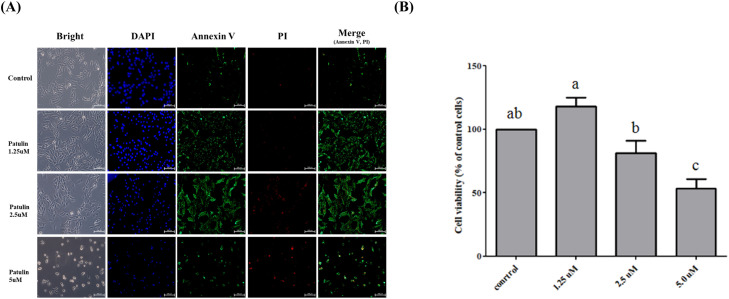
**Dose-dependent effect of patulin on quail myoblast QM7 cells.** (A) Morphology of QM7 cells and results of Annexin V and PI staining of QM7 cells under patulin treatment. (B) Proliferation analysis of QM7 myoblasts treated with various doses of patulin. Data are expressed as the mean ± SD (n = 3). Statistical significance was determined using one-way ANOVA. ^a-d^ Depict the results of statistical analysis (one-way ANOVA and Duncan test); values followed by the same letter in a Duncan grouping are not significantly different; the subscript number and letter color correspond to the chart legend.

To establish the optimal treatment conditions of patulin, QM7 cells were exposed to various concentrations of patulin (0, 1.25, 2.5, and 5 µM) for 24 hours (Fig. 1). Patulin treatment resulted in a dose-dependent decrease in cell viability, and significant cytotoxicity was observed for most concentrations (Fig. 1A). However, notably, cell viability analysis at 1.25 µM of patulin revealed an unexpected increase in QM7 cells (Fig. 1B). Furthermore, the results of annexin V/propidium iodide (**PI**) staining showed a distinctly elevated expression in PI at 2.5 µM of patulin among the various treatment concentrations, indicating severe damage to the cell membrane. Collectively, we determined that 2.5 µM patulin was the appropriate concentration to induce damage in muscle and selected it for subsequent experiments.

**Fig. 2:**
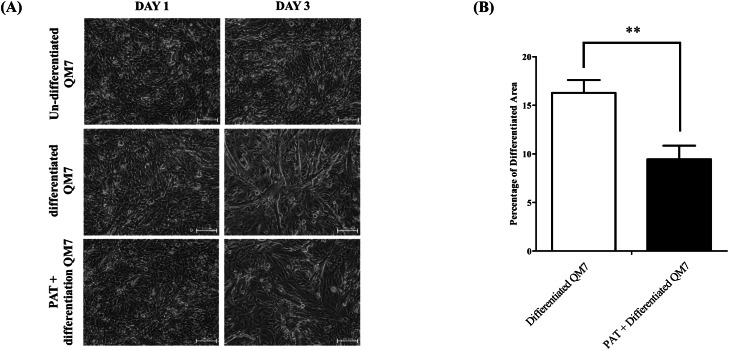
**Effects of patulin on muscle differentiation.** (A) Morphology of myoblasts and myotubes following patulin treatment. (B) Differentiation area analysis of patulin-treated QM7 cells. Differentiation analysis was conducted using the ImageJ software. Data are expressed as the mean ± SD (n = 3). * p < 0.1, ** p < 0.05, *** p < 0.01, **** p < 0.001 calculated using unpaired two-tailed Student’s t-test.

To investigate the effects of patulin on muscle differentiation, QM7 cells were induced to differentiate under patulin treatment. Starting from differentiation day 0, the differentiation medium was changed daily, and patulin treatment was continuously conducted. No difference was observed between the untreated group and patulin-treated groups on differentiation day 1. However, a significant difference in muscle differentiation was observed between the untreated group and patulin-treated groups on differentiation day 3. The patulin-treated group showed reduced myotube formation compared to the untreated group, and the reduction in myotube formation correlated with a significant increase in cell death, thereby impairing cell fusion and subsequent myotube development (Fig. 2A). Compared with the untreated group, the patulin-treated group showed significantly thinner myotube diameters and impaired cell fusion. Furthermore, differentiation area analysis was conducted using ImageJ software to quantify these morphological changes. The analysis suggested that untreated QM7 cells occupied an average of 17 % of the total area undergoing muscle differentiation, whereas the patulin-treated group occupied an average of 12 % (Fig. 2B).

### Cytotoxicity analysis of candidate microbiome

**Fig. 3:**
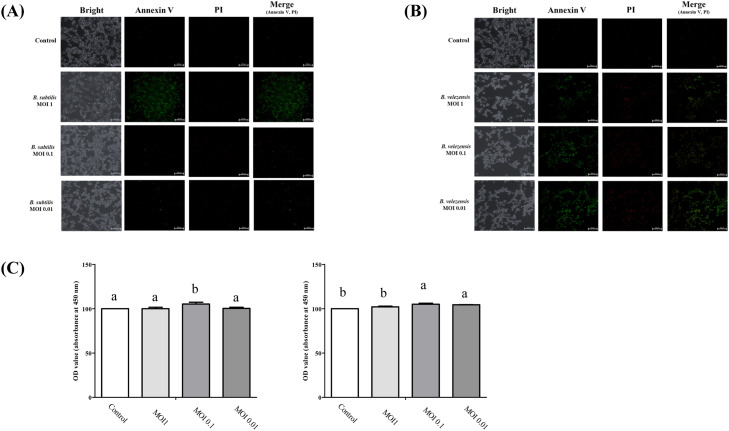
**Cytotoxicity analysis of candidate microbiome, *B. subtilis* and *B. velezensis*, in QM7 cells.** (A) Annexin V and PI staining under (A) *B. subtilis* and (B) *B. velezensis* treatment. (C) Proliferation analysis of QM7 myoblasts in the candidate microbiome (left panel, *B. subtilis*; right panel, *B. velezensis*).

In a previous study, we isolated a candidate microbiome for veterinary drugs and verified its anti-inflammatory effects through functional evaluation (Choi et al., 2025). In this study, we investigated the muscle damage reduction effects of mycotoxins using *Bacillus subtilis* and *Bacillus velezensis* microbiome strains derived from our previous study. To verify the appropriate treatment concentrations for the candidate strains, we conducted a cell viability assay on QM7 myoblasts with varying concentrations of the candidate strains (Fig. 3). For *B. subtilis*, annexin V/ PI staining revealed significant cell death at a multiplicity of infection (**MOI**) of 1; however, cytotoxicity was not high at MOIs of 0.1 and 0.01(Fig. 3A). Furthermore, cell proliferation analysis demonstrated a decrease in the total cell number at an MOI of 1, whereas MOI 0.1 and 0.01 showed an increase (Fig. 3C). Treatment with *B. velezensis* at an MOI of 1 resulted in clear evidence of cell death and a substantial reduction in the total cell number. Interestingly, the extent of cell death was diminished at lower concentrations (MOIs of 0.1 and 0.01) compared with the MOI 1 treatment (Fig. 3B). Therefore, based on these results, we concluded that an MOI of 0.1 was appropriate for both *B. subtilis and B. velezensis.*

### Candidate microbiome recovers myotube formation and morphology

**Fig. 4:**
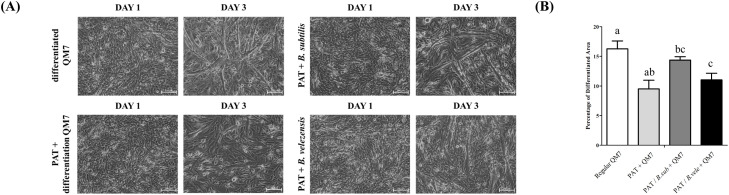
**Evaluation of patulin-induced muscle damage reduction by microbiome (*B. subtilis* and *B. velezensis*).** (A) Morphology of myoblasts and myotubes after the microbiome treatment of patulin-treated QM7 cells. (B) Differentiation area analysis of microbiome and patulin-treated QM7 cells. Data are expressed as the mean ± SD (n = 3). Statistical significance was determined using one-way ANOVA. ^a-d^ Depict the results of statistical analysis (one-way ANOVA and Duncan test); values followed by the same letter in a Duncan grouping are not significantly different; the subscript number and letter color correspond to the chart legend.

Based on the finding that patulin impairs muscle differentiation (Fig. 2), we next investigated the potential of the candidate strains to recover this damage. The obtained strains were co-cultured with patulin-treated QM7 muscle cells, and the differentiation medium was changed every 24 hours from day 0 to day 3, similar to the previous experiment. Patulin and the candidate strains were co-treated during medium change without pre-incubation. In the patulin-treated group, the total cell number was reduced, and muscle differentiation was significantly decreased (Fig. 4A). However, in the microbiome-treated sample group, muscle differentiation significantly recovered compared to that in the patulin-treated group (Fig. 4A, right panel). In the *B. subtilis*-treated group, the fusion of myoblasts increased, leading to the generation of numerous myotubes. Although not fully recovered compared to the untreated group, a noticeable improvement in muscle differentiation was observed. Compared with the *B. subtilis*-treated group, the *B. velezensis*-treated group did not demonstrate a similar level of myoblast fusion; however, an increase in the total cell number was observed, along with a slight increase in the differentiated area. To quantify the extent of myotube formation resulting from these distinct recovery patterns, the myotube area was analyzed using ImageJ software (Fig. 4B). The untreated group showed a myotube area of approximately 16 %. However, as in the previous experiment, the patulin-treated group showed a reduced myotube area of approximately 9.5 %, indicating decreased differentiation. Furthermore, the *B. subtilis*-treated group showed a myotube area of approximately 14.5 %, showing that differentiation had not fully recovered to the levels of the untreated group. Whereas, the *B. velezensis*-treated group exhibited a 12 % of myotube area.

### Candidate microbiome regulates gene expression related to apoptosis, protein degradation, and myogenesis

**Fig. 5:**
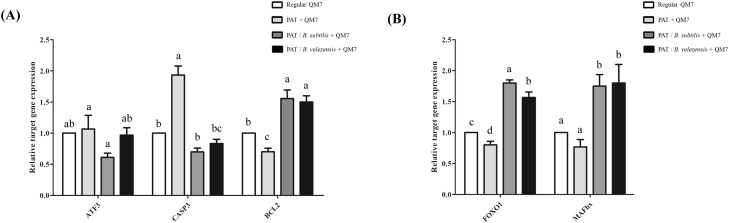
**Modulatory effects of patulin-microbiome co-treatment on cellular stress, apoptosis, and protein degradation pathways during myoblast differentiation.** (A) Expression profile of stress and apoptosis marker genes and (B) protein degradation marker genes after patulin-microbiome co-treatment on day 3 of differentiation. The relative expression for each gene was normalized to that of *GAPDH* and calculated using the $2^{-\Delta \Delta \text{Ct}}$ method (mean ± SD of triplicate experiments; two-tailed student t-test). Data are expressed as the mean ± SD (n = 3). Statistical significance was determined using one-way ANOVA. ^a-d^ Depict the results of statistical analysis (one-way ANOVA and Duncan test); values followed by the same letter in a Duncan grouping are not significantly different; the subscript number and letter color correspond to the chart legend. *ATF3*, Activating Transcription Factor 3; *CASP3*, Caspase 3; *BCL2*, B-cell lymphoma 2; *FOXO1*, Forkhead box protein O1; *FBXO32*, F-box protein 32 (also known as *Atrogin-1/MAFbx*); *GAPDH*, glyceraldehyde 3-phosphate dehydrogenase.

**Fig. 6:**
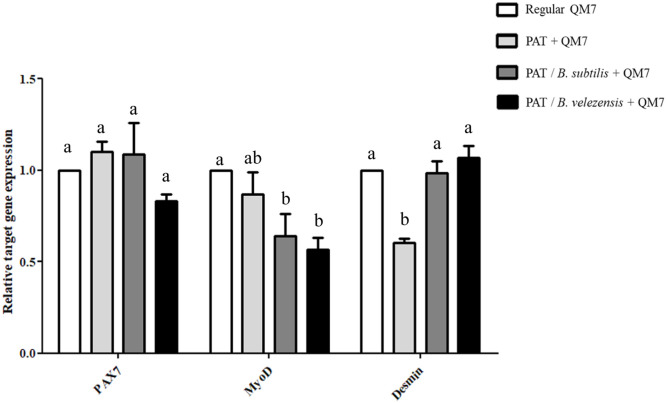
Expression profile of myogenesis marker genes under patulin-microbiome co-treatment under differentiation.

To investigate the molecular mechanisms underlying the protective effects of the candidate microbiome, we performed qPCR to assess the expression of key genes involved in apoptosis, protein degradation (Fig. 5), and myogenesis (Fig. 6). First, we investigated the expression of genes associated with cellular stress and apoptosis (Fig. 5A). In the patulin-treated group, the expression of the cellular stress marker Activating Transcription Factor 3 (***ATF3***) was slightly increased, although this change was not statistically significant. In contrast, a significant upregulation was observed in the pro-apoptotic marker Caspase 3 (***CASP3***), and the expression of the anti-apoptotic marker B-cell lymphoma 2 (***BCL2***) was significantly suppressed. Co-treatment with the candidate microbiomes altered these expression patterns. For *ATF3*, co-treatment with *B. subtilis* reduced its expression, whereas co-treatment with *B. velezensis* did not show a similar effect. However, both *B. subtilis* and *B. velezensis* significantly reduced the patulin-induced expression of *CASP3*. Furthermore, both strains caused a significant upregulation of *BCL2*, elevating its expression to a level approximately 1.5-fold higher than that of the untreated control group. Next, we analyzed the expression of genes involved in the muscle protein degradation pathway to further investigate the protective mechanisms (Fig. 5B). Interestingly, the patulin-treated group showed a slight but significant decrease in the expression of both Forkhead box protein O1 (***FOXO1***) and F-box protein 32 (***FBXO32***) compared to the control group. However, the expression of both genes was significantly increased in the patulin and microbiome co-treatment groups compared to the patulin-only group. Finally, to evaluate the effects on the myogenic differentiation program, we assessed the expression of key myogenic markers (Fig. 6). The expression of paired box 7 (***PAX7***) showed no statistically significant differences across any of the treatment groups. The expression of myogenic differentiation 1 (***MyoD***) was not significantly altered by patulin treatment alone; however, a significant decrease was observed in patulin and microbiome co-treatment groups. In stark contrast, a significant effect was observed on the expression of *Desmin*. Patulin treatment significantly reduced *Desmin* expression, but co-treatment with both *B. subtilis* and *B. velezensis* restored its expression to a level comparable to that of the control group. This result is consistent with the morphological recovery of myotubes observed previously (Fig. 4).

The relative expression for each gene was normalized to that of *GAPDH* and calculated using the $2^{-\Delta \Delta \text{Ct}}$ method (mean ± SD of triplicate experiments; two-tailed student t-test). Data are expressed as the mean ± SD (n = 3). Statistical significance was determined using one-way ANOVA. ^a-d^ Depict the results of statistical analysis (one-way ANOVA and Duncan test); values followed by the same letter in a Duncan grouping are not significantly different; the subscript number and letter color correspond to the chart legend. *PAX7*, Paired Box 7; *MyoD*, Myogenic Differentiation 1; *GAPDH*, glyceraldehyde 3-phosphate dehydrogenase.

## Discussion

Several studies have indicated the potential for mycotoxins to affect muscle tissues by regulating diverse mechanisms such as mitochondrial dysfunction, alterations in sphingolipid metabolism, or changes in muscle fiber types (Guerre et al., 2022; Li et al., 2025). However, the roles of mycotoxins in muscle differentiation have not been extensively explored. Previous research has demonstrated that certain mycotoxins, such as deoxynivalenol (**DON**), can directly impair myogenesis by reducing cell viability and the expression of cytoskeletal proteins, which is consistent with the general muscle damage observed in our study (Jia et al., 2021). In addition, previous studies have indicated that certain mycotoxins, such as zearalenone, can induce oxidative stress and damage in skeletal muscle cells, a process often associated with apoptosis (Li et al., 2025). Furthermore, the dysregulation of key signaling pathways controlling protein synthesis and degradation, such as the Akt/mTOR pathway, has been reported for toxins like verrucarin A, although these effects were primarily observed in non-muscle cell types (Liu et al., 2016). While these studies establish that mycotoxins can trigger cell death and interfere with cellular metabolism, the precise molecular signature of patulin's toxicity during myoblast differentiation has not been well characterized.

The results of the present study indicate that patulin induces myotoxicity through a distinct mechanism. While patulin treatment clearly induced apoptosis, evidenced by the increased expression of the stress-responsive transcription factor *ATF3* (Ku and Cheng, 2020) and the executioner caspase *CASP3* (Fig. 5A), it simultaneously suppressed the expression of the key anti-apoptotic factor *BCL2* (Adams & Cory, 2018) and the master regulators of muscle protein degradation, *FOXO1* and *FBXO32* (Fig. 5A, 5B). The activation of the ubiquitin-proteasome system via *FOXO1* is known to be an active, energy-dependent process central to muscle protein degradation (Sandri et al., 2004). The observed suppression of this pathway, despite lethal cellular damage, suggests that patulin's primary mode of attack is not an upregulation of genes controlling protein degradation. Instead, these results strongly suggest that the acute nature of patulin toxicity induces a global transcriptional suppression, a state where the expression of most adaptive-response genes is shut down. This phenomenon is consistent with observations in sepsis models, where transcriptome sequencing analysis has revealed a global dysregulation of gene expression, including alterations in protein degradation-related genes (Yan et al., 2024).

In response to this patulin-induced cellular paralysis, the primary protective mechanism appeared to be the potentiation of cellular survival signaling. A key indicator of this was the expression of *BCL2,* a crucial anti-apoptotic protein that maintains mitochondrial integrity and prevents the activation of caspases (Adams & Cory, 2018). Indeed, several studies have demonstrated that probiotics can exert cytoprotective effects by modulating the PI3K/Akt pathway, a master regulator of cell survival (Manning, 2017). For example, a soluble protein derived from *Lactobacillus rhamnosus* was shown to protect intestinal epithelial cells from cytokine-induced apoptosis by activating Akt and subsequently upregulating *BCL2* (Yan, 2012).

Consistent with these pro-survival mechanisms, our results showed that by upregulating *BCL2* to a level 1.5-fold higher than the control (Fig. 5A), the strains established a powerful pro-survival state. This upregulation of *BCL2* was accompanied by a significant reduction in the expression of the upstream stress sensor *ATF3* (Ku and Cheng, 2020) and the final executioner caspase, *CASP3* (Fig. 5A). Collectively, these results indicate that the candidate microbiomes actively fortify the cell's anti-apoptotic defenses as a primary strategy to counteract patulin-induced cytotoxicity

Once cell survival was secured, the recovery process entered its next stage. This stage revealed the most novel and paradoxical finding of this study, which was the active upregulation of the muscle protein degradation pathway. The expression of *FOXO1*, a master switch for the ubiquitin-proteasome system, and its target gene *FBXO32* was significantly increased by the candidate strains.

We interpret this upregulation not as a sign of further damage, but as an essential preparatory step for cellular rebuilding. This interpretation is strongly supported by our concurrent observations of suppressed apoptosis (Fig. 5A), evidenced by decreased *CASP3* and increased *BCL2* expression, and the successful restoration of the structural protein Desmin (Fig. 6). A damaging process would not be expected to be accompanied by such potent pro-survival and pro-maturation signals. Instead, we propose that this represents a necessary phase for the removal of damaged cellular components to prevent their toxic accumulation and to provide raw materials for new synthesis. The necessity of removing damaged cellular components for successful tissue repair is well-supported by the role of quality control systems, such as the ubiquitin-proteasome pathway, in maintaining muscle homeostasis (Khalil, 2018). Therefore, the upregulation of the protein degradation pathway by the candidate strains can be interpreted as a crucial mechanism to clear molecular wreckage from patulin's attack, thereby paving the way for the final maturation stage.

With the cellular environment stabilized and cleansed, the final stage of the recovery process involved the completion of myotube maturation (Fig. 6). This was evidenced by the expression patterns of key myogenic markers, which are sequentially expressed to drive differentiation. The expression of the early marker *MyoD*, a key myogenic regulatory factor (MRF) that activates the differentiation program (Tapscott, 2005), was significantly decreased in the microbiome-treated sample groups. Concurrently, the expression of the late structural protein Desmin, which contributes to the structural integrity of mature muscle cells (Ganassi et al., 2020), was completely restored to the level of the control group.

This inverse relationship between the early (*MyoD*) and late (*Desmin*) differentiation markers suggests an acceleration of the myogenic program. The temporal expression pattern of MRFs, where *MyoD* initiates the process and is later downregulated as late-stage genes become active, is a well-established hallmark of efficient myogenesis (Olguin & Olwin, 2004). Taken together, this expression profile suggests that the co-treated cells were not stalled in an early differentiation phase. Instead, by the day 3 timepoint, they had likely already progressed efficiently through the initial *MyoD*-dependent stages and had robustly entered the final maturation phase, which is characterized by high levels of structural proteins like Desmin. Therefore, these results lead us to conclude that the candidate strains do not simply recover the final differentiated state, but fundamentally enhance the kinetics and efficiency of the entire myogenic process.

*Bacillus* species, such as *B. subtilis* and *B. velezensis*, are widely used in diverse sectors including agriculture and medicine. While studies on the effects of these *Bacillus* species on muscle tissue are limited, some research has shown that *B. subtilis* can aid muscle recovery through inflammation regulation (Townsend et al., 2018) or alter muscle fiber characteristics in pigs (Liu et al., 2024). Similarly, indirect evidence suggests a potential role for *B. velezensis* in improving growth performance (Yu et al., 2025).

Our findings expand on this existing knowledge by providing a direct cellular mechanism for these effects. Our results indicate that the candidate *Bacillus* strains counteract mycotoxin-induced damage by directly modulating intrinsic cellular pathways governing survival, protein degradation, and differentiation, rather than through the previously suggested systemic effects like anti-inflammation (Townsend et al., 2018). Specifically, the observation of a regulated protein degradation phase followed by an accelerated maturation process represents a novel mechanism for probiotic-mediated tissue repair. These molecular findings provide a potential cellular-level explanation for the positive effects on muscle tissue observed in previous animal studies (Liu et al., 2024; Yu et al., 2025) and suggest a more direct and complex role for the tested probiotics in promoting muscle health.

In this study, we investigated the potential of *B. subtilis* and *B. velezensis* to counteract mycotoxin-induced muscle damage. The restoration of Desmin expression and myotube morphology provided strong evidence for a restorative effect at the cellular level. However, as this study was conducted using an in vitro model, a crucial next step will be to validate these protective effects in an in vivo animal model, which would also allow for the assessment of the functional maturity of the recovered muscle tissue. In conclusion, this study identified a novel mechanism of patulin-induced myotoxicity characterized by a global transcriptional suppression. Furthermore, we demonstrated that *B. subtilis* and *B. velezensis* employ a multi-stage recovery program involving cellular fortification, constructive degradation, and accelerated maturation. These findings suggest the significant potential of these *Bacillus* strains as therapeutic agents against mycotoxin-induced muscle damage and indicate their role as potent modulators of cellular recovery pathways.

## CRediT authorship contribution statement

**Jeong woong Park:** Writing – review & editing, Writing – original draft, Investigation, Formal analysis, Conceptualization. **Hana Kim:** Software, Data curation. **Seon-Ae Choi:** Validation. **Hak Kyo Lee:** Writing – review & editing, Supervision, Project administration. **Dong Hyun Shin:** Writing – review & editing, Supervision, Project administration.

## Disclosures

The authors declare that they have no known competing financial interests or personal relationships that could have appeared to influence the work reported in this paper.
